# Neuroplastin exerts antiepileptic effects through binding to the α1 subunit of GABA type A receptors to inhibit the internalization of the receptors

**DOI:** 10.1186/s12967-023-04596-4

**Published:** 2023-10-09

**Authors:** Sijun Li, Xing Wei, Hongmi Huang, Lin Ye, Meigang Ma, Lanfeng Sun, Yuling Lu, Yuan Wu

**Affiliations:** Department of Neurology, The First Affiliated Hospital of Guangxi Medical University, Guangxi Medical University, Shuangyong Road No.6, Nanning, Guangxi China

**Keywords:** Seizures, γ-aminobutyric type A acid receptors, Neuroplastin, Internalization

## Abstract

**Background:**

Seizures are associated with a decrease in γ-aminobutyric type A acid receptors (GABAaRs) on the neuronal surface, which may be regulated by enhanced internalization of GABAaRs. When interactions between GABAaR subunit α-1 (GABRA1) and postsynaptic scaffold proteins are weakened, the α1-containing GABAaRs leave the postsynaptic membrane and are internalized. Previous evidence suggested that neuroplastin (NPTN) promotes the localization of GABRA1 on the postsynaptic membrane. However, the association between NPTN and GABRA1 in seizures and its effect on the internalization of α1-containing GABAaRs on the neuronal surface has not been studied before.

**Methods:**

An in vitro seizure model was constructed using magnesium-free extracellular fluid, and an in vivo model of status epilepticus (SE) was constructed using pentylenetetrazole (PTZ). Additionally, in vitro and in vivo NPTN-overexpression models were constructed. Electrophysiological recordings and internalization assays were performed to evaluate the action potentials and miniature inhibitory postsynaptic currents of neurons, as well as the intracellular accumulation ratio of α1-containing GABAaRs in neurons. Western blot analysis was performed to detect the expression of GABRA1 and NPTN both in vitro and in vivo. Immunofluorescence co-localization analysis and co-immunoprecipitation were performed to evaluate the interaction between GABRA1 and NPTN.

**Results:**

The expression of GABRA1 was found to be decreased on the neuronal surface both in vivo and in vitro seizure models. In the in vitro seizure model, α1-containing GABAaRs showed increased internalization. NPTN expression was found to be positively correlated with GABRA1 expression on the neuronal surface both in vivo and in vitro seizure models. In addition, NPTN overexpression alleviated seizures and NPTN was shown to bind to GABRA1 to form protein complexes that can be disrupted during seizures in both in vivo and in vitro models. Furthermore, NPTN was found to inhibit the internalization of α1-containing GABAaRs in the in vitro seizure model.

**Conclusion:**

Our findings provide evidence that NPTN may exert antiepileptic effects by binding to GABRA1 to inhibit the internalization of α1-containing GABAaRs.

**Graphical Abstract:**

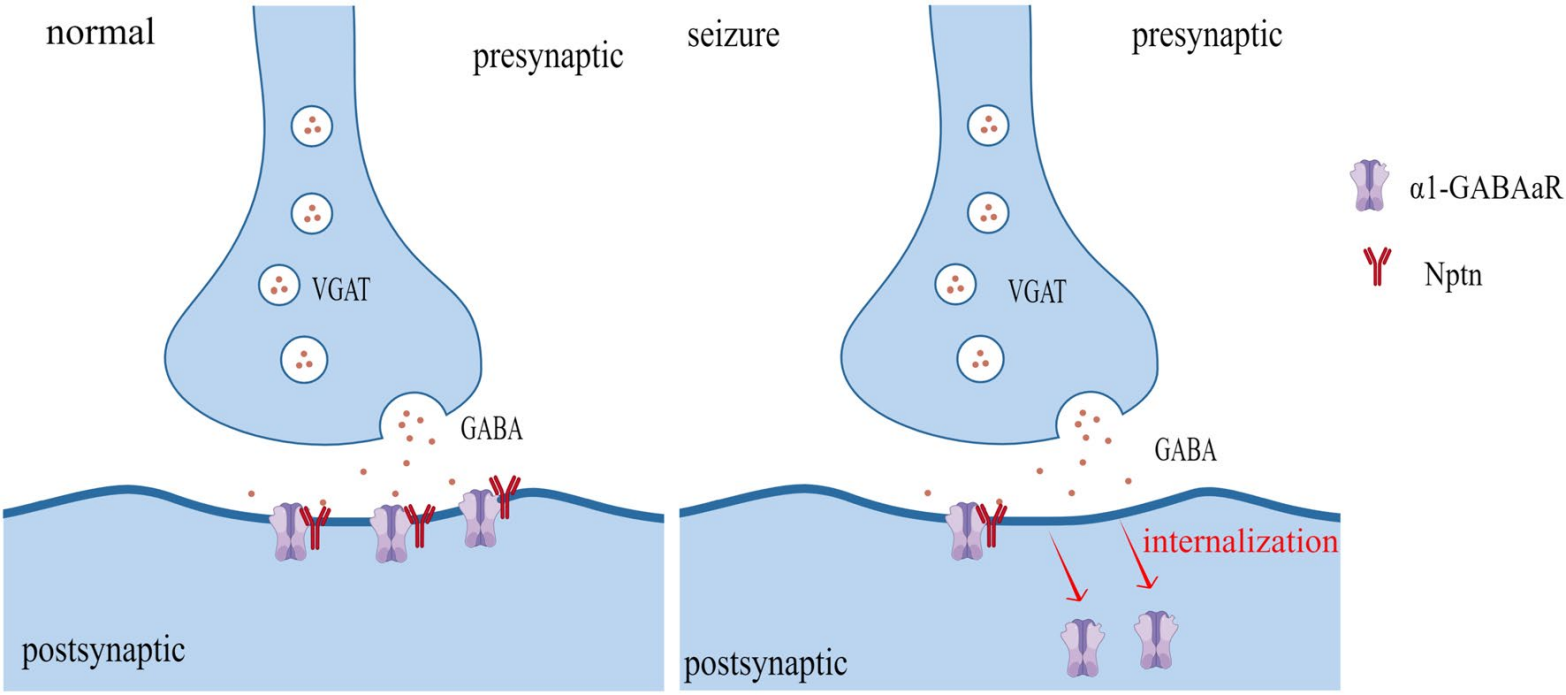

**Supplementary Information:**

The online version contains supplementary material available at 10.1186/s12967-023-04596-4.

## Background

Acute seizures are associated with an imbalance between neuronal excitation and inhibition (E/I imbalance) that leads to synchronized neuronal discharge [1]. The most serious consequence of seizures is status epilepticus (SE), which has a mortality rate as high as 34% [2, 3]. E/I imbalance may be attributed to strengthened excitatory synapses and/or weakened inhibitory synapses [4]. There are evidences that increases in excitatory neurotransmitters [5] and/or glutamate receptors on the neuronal membrane strengthens excitatory synapses [6], whereas decreases in inhibitory neurotransmitters [7] and/or γ-aminobutyric type A acid receptors (GABAaRs) on the neuronal membrane weakens inhibitory synapses [8]. Compared with the reduced release of GABA/inhibitory neurotransmitters in neurons, the decrease in GABAaRs on the neuronal surface is more closely related to acute seizures [9].

GABAaRs are fast-gated chloride ion channels comprising five subunits that are also the active sites of benzodiazepines [10, 11]. Activation of GABAaRs promotes a rapid influx of chloride ions, resulting in a postsynaptic inhibitory effect that mediates the rapid response of the neural inhibitory mechanism. These events are crucial for terminating epileptic seizures [12]. Eight types of GABAaRs have been previously described [13]. The most abundant subtype (α1β2γ2) comprises α1, β2, and γ2 subunits [14, 15]. The reduced number of GABAaRs on the neuronal surface is related to the enhanced internalization of GABAaRs, which is the entry of the receptors from the cell membrane into the intracellular compartments [16]. GABAaRs accumulate at the postsynaptic membrane through their interaction with postsynaptic scaffold proteins that promotes the stabilization of GABAaRs on the neuronal surface and inhibits receptor internalization [17]. When the interactions between GABAaRs and postsynaptic scaffold proteins are weakened, GABAaRs leave the postsynaptic membrane and are internalized. However, it remains unclear whether the internalization of GABAaRs is involved in seizures.

The α subunits of GABAaRs are the most abundant subunits of these receptors, and the α1 subunit (GABRA1) in particular plays a key role in terminating seizures [18]. It is known that these α subunits can bind to the cell surface scaffold proteins Rho guanine nucleotide exchange factor 9 (ARHGEF9) and gephyrin (GPN) to maintain the number of GABAaRs on the cell membrane [19, 20]. However, the binding ability of GABRA1 to ARHGEF9 and GPN is weak [21, 22]. Given the significant number of GABRA1, the mechanism that governs the decrease of α1-containing GABAaRs on the neuron surface in seizures is not well understood. Postsynaptic scaffold proteins may be involved in this mechanism. Nevertheless, the postsynaptic scaffold proteins that significantly interact with GABRA1 remain unclear.

Neuroplastin (NPTN), as a member of the Ig superfamily, plays a role as a cell adhesion molecule (CAM) [23]. NPTN, which is abundant in the brain, plays an important role in many biological functions, including spatial learning, hearing and cognitive function, immune inflammation, energy metabolism and nerve growth, through the mechanisms of regulation of Ca2 + concentration, endoplasmic reticulum stress, monocarboxylate transporter(MCT2) and tumor necrosis factor receptor-associated factor 6 (TRAF6) and neurite outgrowth [24–30].In addition, NPTN also produces a marked effect of regulating neuronal electrophysiology. Vemula et al. suggested that overexpression of NPTN promotes spinogenesis which affect the general electrophysiological activity of the neuronal network in vitro [29]. Besides, NPTN can influence the synaptic excitability by regulating neurotransmitter receptors to affect the neuronal electrophysiology. Previous studies have shown that NPTN can influence excitatory synapses by regulating AMPA and NMDA receptors [23, 31, 32]. Overexpression of NPTN would result in increased levels of Plasma Membrane Ca2 + -ATPases (PMCA) [24], which interact functionally with NMDAR and AMPAR in hippocampal neurons [31, 32]. Herrera-Molina et al. have further shown that NPTN can influence inhibitory synapses by affecting GABAaRs mobility [33]. Sarto-Jackson et al. demonstrated that NPTN could enhance stability of GABAaRs by interacting with the receptors, which may be an important mechanism for regulating inhibitory synapses [34].Taken together, the results suggest that NPTN may play a role in seizures. However, the mechanisms of NPTN in seizures has not been studied before.

Therefore, we hypothesized that NPTN might be involved in seizures and inhibit seizures by affecting GABRA1. In vitro and in vivo seizure models were constructed to identify the levels of NPTN and GABRA1 after seizures and thereby investigated the potential mechanism.

## Methods

### Animals

Twenty-six adult male Sprague–Dawley (SD) rats (200 g–250 g) obtained from the Animal Experiment Center of Guangxi Medical University, China, were used for experiments. Adult male SD rats were fed under the following conditions: 22–26 ℃, 50–60% humidity, light–dark cycle at 12:12, and light on at 8:00, with 6 rats per cage. The rats had ad libitum access to food and water. Newborn rats (24 h-old) were used for neuronal cell culture. The animal ethics review followed the Guiding Opinions on the Treatment of Laboratory Animals issued by the Ministry of Science and Technology of the People's Republic of China and Laboratory Animal Guidelines for Ethical Review of Animal Welfare issued by the National Standard GB/T35892-2018 of the People's Republic of China. The minimum sample size of experimental animals was calculated using statistical methods based on pre-experimental data to reduce the number of animals while ensuring as much comparability of the experimental data as possible. At the end of the experiment, the animals were deeply anesthetized (3% sodium pentobarbital) and sacrificed to alleviate pain.

### Primary neuron culture and transfection

Before primary neuron culture, 35-mm tissue culture petri dishes were incubated with poly-D-lysine (Gibco, A3890401) in 37 ℃ for 10 h. Then, following two media were prepared: serum-neurobasal medium = 88% GibcoDulbecco's Modified Eagle Medium: F-12(DMEM/F12)(Gibco, A4192001) + 1% Glutamax (Gibco, 35050061) + 1% penicillin–streptomycin (Gibco, 15140–122) + 10% serum (Gibco, A3160902); serum-free-neurobasal medium = 96% Neurobasal™-A (Gibco, 10888022) + 1% Glutamax (Gibco, 35050061) + 1% penicillin–streptomycin (Gibco, 15,140–122) + 2% B27 supplement (Gibco, 17504044). Hippocampi from the anesthetized newborn rats (24 h-old) were dissected in ice-cold Hank’s buffered salt solution (ThermoFisher, #14185052) with 10 mM 2-[4-(2-hydroxyethyl)piperazin-1-yl]ethanesulfonic acid (HEPES) and subsequently snipped and incubated with 0.125% trypsin–EDTA (Gibco, A4192001) at 37℃ for 12 min. Cells were then washed once with serum-neurobasal medium to terminate trypsin–EDTA treatment. After filtering through a 40-mm nylon mesh strainer to remove non-dissociated tissue, hippocampal neurons were counted on a hemocytometer and plated in dishes with 2 mL of serum-neurobasal medium. After 10 h, the serum-neurobasal medium was removed and replaced with a serum-free neurobasal medium.

Lentiviruses (LVs) were purchased from Sangon Biotechnology (Shanghai) Co., Ltd., including LV negative control (LV-NC, titer: 3.83 × 10^9^ TU/mL) and LV overexpressing neuroplastin (LV-OE-NPTN; titer: 1.53 × 10^9^ TU/mL). In the LV-OE-NPTN group, we loaded the cDNA of the transcript NM_019380 of the target gene neuroplastin into the LV vector constructions with hUbC promoter and this transcript coding neuroplastin isoform 2 precursor (NP_062253) which contains 393 amino acids. Neurons cultured for 3 days were transfected with LV-NC or LV-OE-NPTN (multiplicity of infection = 5). After 72 h, the cell morphology was examined using a Nikon microscope. NPTN expression was detected using western blotting.

### Mg^2+^-Free model in vitro

After 15 days of culture, neurons were exposed to Mg^2+^-free extracellular fluid for 3 h, which led to synchronized discharges [35]. On day 15 of culture, neurons were divided into control and Mg^2+^-free extracellular fluid groups. The neurons in the control group were exposed to a normal extracellular solution comprising (in mM) sodium chlorid (NaCl) (145), potassium chloride (KCl) (2.5), HEPES (10), calcium chloride (CaCl_2_^)^ (2), glucose (10), glycine (0.002), and magnesium chloride (MgCl_2_^)^ (1) for 3 h. In contrast, the neurons in the Mg^2+^-free extracellular fluid group were exposed to a Mg^2+^-free extracellular solution comprising (in mM) NaCl (145), KCl (2.5), HEPES (10), CaCl_2_ (2), glucose (10), and glycine (0.002) for 3 h.

Action potentials (APs) were obtained by whole-cell patch-clamp recording using a Digidata 1550 B patch-clamp amplifier, Axon Digidata 1550 B 16-bit data acquisition system, and pClamp 10.7 data acquisition software. All recordings were performed in extracellular recording solution containing (in mM) NaCl (122), KCl (2), HEPES (25), CaCl_2_ (2), MgCl_2_ (4), and glucose (10), with a pH of 7.4. Recordings were obtained using patch electrodes (resistance of 3–6 MΩ) filled with an intracellular solution containing (in mM) KCl (110), NaCl (1), ethylene glycol tetraacetic acid (EGTA) (2), HEPES (25), adenosine 5′-triphosphate magnesium salt (Mg-ATP) (4), guanosine 5'-(disodium dihydrogen triphosphate) (Na_2-_GTP) (0.3), and phosphocreatine (10), with a pH of 7.3. Whole-cell recording was used and action potentials (APs) were recorded with a clamping voltage of -70 mV. Miniature inhibitory postsynaptic currents (mIPSCs), which are mainly produced by postsynaptic GABAaRs [36], were recorded as described by Wyrembek et al. [37]. All recordings were performed in extracellular recording solution containing (in mM) NaCl (137), KCl (5), CaCl_2_ (2), MgCl_2_ (1), glucose (20) and HEPES (10), with a pH of 7.2 with NaOH. The intracellular solution was composed of (in mM) cesium chloride (CsCl) (137), CaCl_2_ (1), MgCl_2_ (2), tetra cesium salt (BAPTA) (11), adenosine triphosphate (ATP) (2), and HEPES (10), with a pH of 7.2 with cesium hydroxide (CsOH). mIPSCs were recorded in the whole-cell configuration in the gap-free mode. All measurements were recorded at 25 ℃. The data were analyzed using Clampfit 10.7 and MiniAnal. In the in vitro model, a single neuron was considered as the experimental unit. Six neurons from each group were selected for the patch-clamp analysis.

### Pentylenetetrazol-induced SE in vivo

SE was induced in rats via intraperitoneal injection of Pentylenetetrazol (PTZ), a non-competitive antagonist of GABAaRs. PTZ (Sigma-Aldrich, St. Louis, MO, USA) was dissolved in physiological saline before injections (10%). The rats were randomly divided into a normal control group (*n* = 6) and a PTZ-induced SE group (*n* = 6). Rats in the SE group were intraperitoneally injected with PTZ at an initial dose of 40 mg/kg, followed by 20 mg/kg every 10 min until SE development [38]. Rats in the control group were treated with physiological saline. The time of PTZ and saline injection was between 10:30 am to 12:30 am. Seizures were classified as follows [39, 40]: stage 0, no seizures; stage 1, ear and facial twitching; stage 2, strong myoclonus but no upright position; stage 3, strong myoclonus with upright position and bilateral forelimb clonus; stage 4, clonic–tonic seizures; and stage 5, generalized clonic–tonic seizures and loss of postural control. SE was defined as stage 4 or higher seizure, lasting at least 30 min with prolonged episodes of seizures interrupted by postictal phases of depression, but without regaining quadruped posture or consciousness [41]. The latent period of each sample was calculated based on the characteristics of an epileptic seizure, that is, the time between the first injection of PTZ and first seizure. The duration of episodes of stage 4 or higher seizure was recorded. Next, the rats were deeply anesthetized with isoflurane and their hippocampi were separated for biochemical experiments. In the in vivo model, a single animal was considered as the experimental unit.

### Adeno-associated viral (AAV) vector construction and stereotaxic injections

AAV vector constructs tagged with green fluorescence were purchased from Sangon Biotech Co., Ltd. (Shanghai), including AAV negative control (AAV-NC, titer: 3.06 × 10^13^ VG/mL) and AAV overexpressing neuroplastin (AAV-OE-NPTN, titer: 7.03 × 10^13^ VG/mL). We loaded the cDNA of the transcript NM_019380 of the target gene neuroplastin into the AAV vector constructions with hSyn promoter in the AAV-OE-NPTN group and this transcript coding neuroplastin isoform 2 precursor (NP_062253) which contains 393 amino acids. The rats were randomly divided into AAV-NC (n = 7) and AAV-OE-NPTN (n = 7) groups. The rats were deeply anesthetized with 3% sodium pentobarbital, placed on a stereotaxic apparatus, and fixed with ear bars and head holders. Each rat received 1 μL of AAV vector (0.2 μL/min) per hippocampus using a 5-μL glass syringe (Hamilton 7633–01) and bevel-tip needle (Hamilton, 30°, 30 gauge, 0.5″). The coordinates for hippocampal injections were established as follows: anterior/posterior(AP): − 3.0 mm, lateral/medial (LM): ± 2.2 mm, dorsal/ventral (DV): − 3.5 mm). Two rats from each group were randomly selected 4 weeks after surgery to evaluate NPTN expression in the hippocampus based on spontaneous fluorescence and expression levels. Five rats from each group were injected intraperitoneally with PTZ at an initial dose of 40 mg/kg, followed by 20 mg/kg every 10 min until the development of SE.

### Electroencephalography (EEG) recordings

The rats were anesthetized with isoflurane for noninvasive fixation. Electrodes were installed on the forehead, temporal cortex, and both forelimbs, noninvasively. EEG signals were recorded using a Nuochen EEG system. Each part was labeled as follows: left frontal Fp1-Avf, right frontal Fp2-Avf, left temporal T3-Avf, and right temporal T4-Avf. EEG signals were collected from each rat for 30 min. Recurrent interictal epileptic discharges, generalized multispike waves and multiple spike and slow-wave complexes recorded by the EEG were the criteria for the success of the model.

### Western blot analysis

Total protein [42] and surface protein (plasma membrane protein) [43] of the neurons and hippocampus were extracted using a Protein Extraction Kit (Invent Biotechnologies, SD-001/SN-002; SM-005). A BCA assay kit (Beyotime, P0012S) was used to evaluate the protein concentrations. Protein samples (20 μg protein/lane) were separated by sodium dodecyl-sulfate polyacrylamide gel electrophoresis (SDS-PAGE) and transferred to a nitrocellulose (NC) membrane (Merck Millipore Ltd.) using a wet transfer cell (Bio-Rad). The NC membrane was blocked and incubated with the primary antibody at 4 ℃ overnight. The primary antibodies used were anti-neuroplastin antibody 1:1000 (Novus, NBP2-01793) (This antibody specificity was verified in Additional file 1), anti-GABA A receptor alpha 1 antibody 1:10000 (Abcam, ab33299), anti-GABA vesicular transporter antibody 1:1000 (Sangon Biotech, D262232), anti-GAPDH antibody 1:10000 (Sangon Biotech, D110016), and anti-ATP1A1 antibody 1:10000 (Proteintech, 14418–1-AP). Subsequently, the NC membrane was incubated with horseradish peroxidase-conjugated AffiniPure goat anti-rabbit immunoglobulin G (H + L) 1:8000 (Proteintech, SA00001-2) and horseradish peroxidase-conjugated Affinipure goat anti-mouse immunoglobulin G (H + L) 1:8000 (Proteintech, SA00001-1) for 1 h at room temperature. Immunoreactive bands were visualized using the Omni-ECL™Femto Light Chemiluminescence Kit (EpiZyme, SQ201) [44] in ChemiScope6000. The ImageJ software was employed to quantify band intensities of the western blot images. Protein levels were determined by normalization to GAPDH (total protein) and/or ATP1A1 (surface protein) loading controls. Each experiment was repeated 3 times.

### Immunofluorescence co-localization analysis

Cultured neurons were fixed with 4% paraformaldehyde for 30 min, permeabilized with 0.1% Triton X-100 for 10 min, and blocked with 5% bovine serum albumin (BSA) for 1 h. The neurons were incubated with anti-GABRA1 antibodies 1:100 (Biorbyt, Orb10677) at 4 ℃ overnight. The neurons were then washed with phosphate-buffered saline PBS and incubated with a red fluorescent secondary antibodies (CST, #8889) at 37 ℃ for 45 min. Other neurons were incubated with anti-NPTN antibodies (Sangon Biotech, D199223) at 37 ℃ for 30 min. The neurons were then washed with PBS and incubated with a green fluorescent secondary antibodies (CST, #4408) at 37 ℃ for 45 min. Lastly, 4',6-diamidino-2-phenylindole (DAPI) staining solution was added and the cells were incubated at room temperature for 3 min.

The paraffin-embedded brain sections were de-waxed using dimethylbenzene. Antigens were retrieved by heating in EDTA in a microwave oven. Subsequently, paraffin sections were blocked with 10% goat serum (Sigma-Aldrich, G9023). Paraffin sections were incubated with anti-GABRA1 antibodies 1:100 (Biorbyt, Orb10677) at 4 ℃ overnight. The sections were washed with PBS and incubated with a red fluorescent secondary antibody (CST, #8889) at 37 ℃ for 45 min. After blocking, the sections were incubated with anti-NPTN antibodies (Sangon Biotech, D199223) at 37 ℃ for 30 min and then washed with PBS. The green fluorescent secondary antibodies (CST, #4408) were used at 37 ℃ for 45 min. Lastly, DAPI staining solution was added and the cells were incubated at room temperature for 3 min. Neurons and hippocampal tissues were examined using an Olympus BX53 fluorescence microscope, and micrographs were obtained. The resolution and imaging conditions of cells were as follows: microscopes, Olympus BX53; objectives, neurons; time of exposure, 50 ms; optic/digital magnification conditions, × 600. The resolution and imaging conditions of tissues were as follows: Olympus BX53; objectives, hippocampal tissues; time of exposure, 250 ms; optic/digital magnification conditions, × 200. ImageJ software was used to analyze the images and measure the Pearson’s correlation coefficient (PCC) (Image-Color-Split Channels; Image-Color-Channels Tool; Analyze-Colocalization-Coloc2). The PCC vary from 0 to 1, the former corresponding to non-overlapping images and the latter reflecting 100% colocalization between both the latter reflecting 100% colocalization between both images [45].

### Co-immunoprecipitation (Co-IP)

Total native hippocampal protein was extracted using a Protein Extraction Kit (Invent Biotechnologies, SD-001/SN-002) [43]. The BCA assay kit (Beyotime, P0012S) was employed to evaluate protein concentrations. An immunoprecipitation Kit (Sangon Biotech, C600689) was used for Co-IP. Native total protein was co-incubated with anti-GABA A receptor alpha 1 antibodies 1:30 (Abcam, ab252430) at 4 ℃ overnight to generate antigen–antibody complex. Protein A/G Plus-Agarose washed using PBS was incubated with the antigen–antibody complex at 4 ℃ overnight. For immunoprecipitation, the agarose beads were washed with the IP buffer. Protein samples were separated by SDS-PAGE and transferred to an NC membrane (Merck Millipore Ltd) by wet transfer cell (Bio-Rad). The primary antibodies used were anti-neuroplastin antibody 1:1000 (Novus, NBP2-01793) and anti-GABA A receptor alpha 1 antibody 1:10000 (Abcam, ab33299). The NC membrane was incubated with horseradish peroxidase-conjugated AffiniPure goat anti-rabbit immunoglobulin G (H + L) 1:8000 (Proteintech, SA00001-2) and horseradish peroxidase-conjugated Affinipure goat anti-mouse immunoglobulin G (H + L) 1:8000 (Proteintech, SA00001-1) for 1 h at room temperature. Immunoreactive bands were visualized using the Omni-ECL™Femto Light Chemiluminescence Kit (EpiZyme, SQ201) [44] in ChemiScope6000. The ImageJ software was employed to quantify band intensities of the western blot images.Pooled quantification of protein immunoprecipitation was employed to evaluate the interaction between NPTN and GABRA1. The IP protein band GABRA1 was used to normalize the pull-down protein levels obtained from Co-IP. The coprecipitated protein levels in each group were then compared.

### Internalization assay

A fluorescence-based GABRA1 internalization assay was performed as described previously [36]. The cultured neurons were incubated with anti-GABRA1 antibodies (Biorbyt, Orb10677) for 1 h at 4 ℃. After brief washing with PBS, neurons were returned to a conditioned medium at 37 ℃ for 90 min to maximize the internalization of GABRA1. Neurons were fixed with 4% paraformaldehyde for 15 min and blocked with 5% BSA for 30 min. Antigen–antibody complexes on the surface were detected by incubating the cells with red fluorescent secondary antibodies (#8889; CST). Neurons were then permeabilized with 0.25% Triton X-100, and intracellular antigen–antibody complexes were detected using green-fluorescent secondary antibodies (Bioss, BA1105). A fluorescence microscope (OLYMPUS BX53) was used to examine the results. Red signals denote receptors that remained on the cell surface, whereas green signals denote internalized surface receptors. The intracellular accumulation ratio of neurons was analyzed to evaluate the cell internalization dynamics (*n* = 9 neurons per group). The GABRA1 intracellular accumulation ratio was defined as the ratio of the internalized fluorescence signal to the total fluorescence signal, that is, the sum of the surface and intracellular signals. GABRA1 intracellular accumulation ratio = red optical density/(red optical density + green optical density).ImageJ software was used to analyze the images. Nine cells selected from each group were used to calculate optical density.

### Statistical analyses

Continuous variables were presented as means ± standard deviations, and categorical variables were presented as frequencies (percentages). An independent sample t-test was used for the comparison between the means of two independent groups. The statistical analysis software used was SPSS version 25.0. Statistical significance level was set at *p* < 0.05.

## Results

### Enhanced internalization of GABAaRs is associated with neural hyperexcitation

**Fig. 1:**
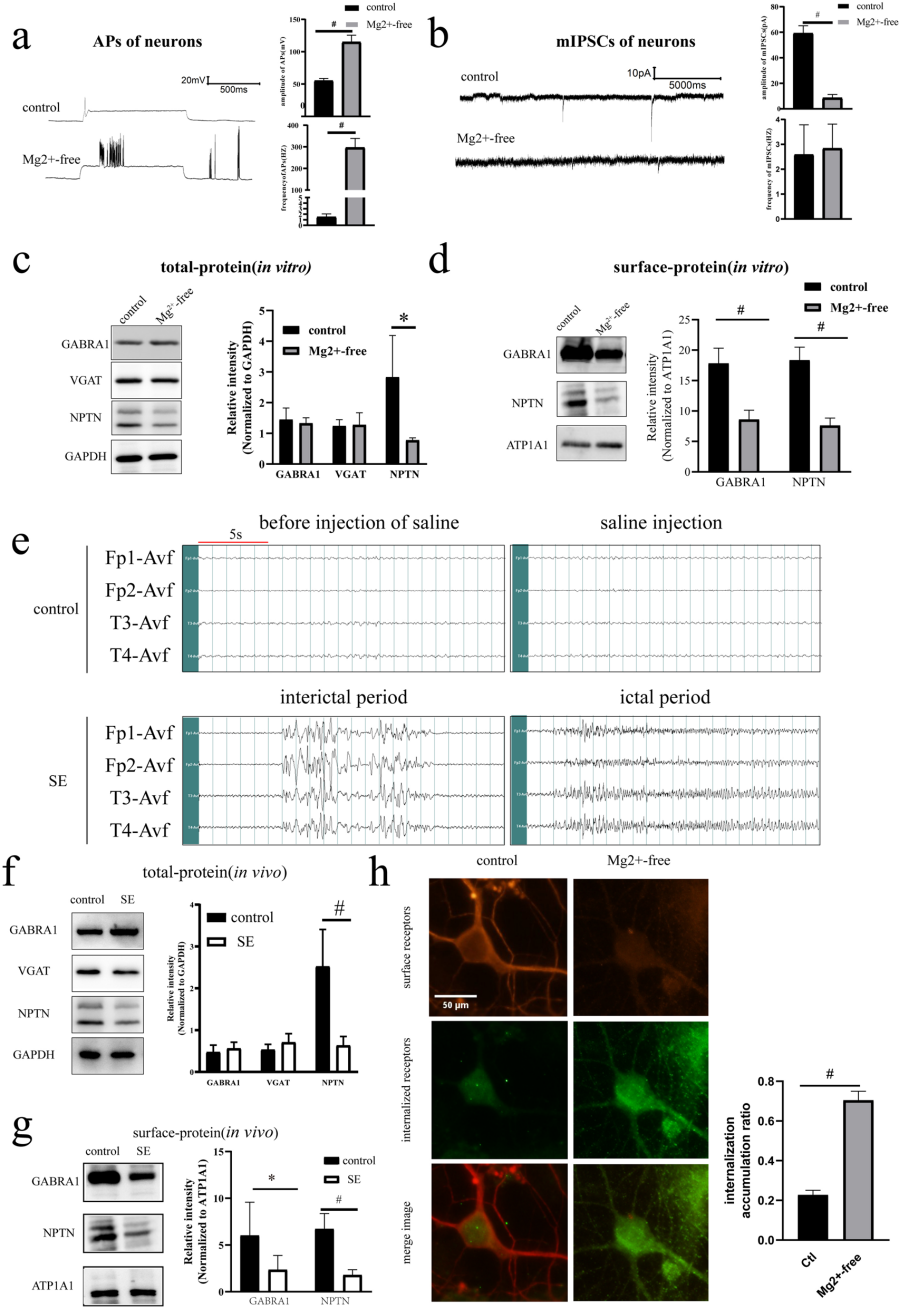
**a** APs of neurons: The amplitude and frequency of neuronal AP in the Mg2 + -free group were higher than those in the control group (*n* = 6 in each group, # *p* < 0.01). **b** mIPSCs of neurons: Compared with that in the control group, the frequency of mIPSCs in the Mg2 + -free group did not change significantly, while the amplitude of mIPSCs in the Mg2 + -free group was decreased (*n* = 6 in each group, * *p* < 0.05). **c** Total protein expression (in vitro): Compared with that in the control group, NPTN expression in the Mg2 + -free group was decreased (*n* = 6 in each group, #*p* < 0.01), while GABRA1 and VGAT expression did not significantly change. **d** Surface protein expression (in vitro): Surface GABRA1 in the Mg2 + -free group was decreased compared with that in the control group (*n* = 5 in each group, #*p* < 0.01). **e** Rat EEG recordings: No significant seizures and epileptic discharges were recorded in the control group. Contrarily, EEG recordings showed recurrent epileptic discharges during the interictal period and generalized multi-spike waves and multi-spike waves complexes during the ictal period in all SE groups. time scale**:** 5 s**. f** Total protein expression (in vivo): Compared with that in the control group, NPTN expression in the SE group was decreased (*n* = 6 in each group, #*p* < 0.01), while the changes in GABRA1 and VGAT expression were not significant. **g** Surface protein expression (in vivo): Surface GABRA1 in the SE group was decreased compared with that in the control group (*n* = 6 in each group, *p < 0.05). **h** Internalization assay (× 600): The intracellular accumulation ratio of neurons in the Mg2 + -free group was increased compared with that of those in the control group (intracellular accumulation ratio_Crtl_ = 0.23 ± 0.02, intracellular accumulation ratio_Mg2+-free_ = 0.70 ± 0.04, *n* = 9 in each group, # *p* < 0.01). All experiments were repeated for 3 times. ^△^Independent t-tests were used for the comparison of means

We constructed in vitro models by exposing neurons to Mg^2+^-free solution. The amplitudes of neuronal APs in the Mg^2+^-free group were significantly higher than those in the control group (*p* < 0.01, Fig. 1a). The frequency of neuronal APs in the Mg^2+^-free group was also significantly higher than those in the control group (*p* < 0.01, Fig. 1a). This suggests an abnormal increase in neuronal excitability after treatment with Mg^2+^-free extracellular solution. To further investigate the inhibitory neural function mediated by GABAaRs, we examined the mIPSCs in the in vitro models. The frequency of mIPSCs in the Mg^2+^-free group did not change significantly compared with that in the control group (*p* > 0.05, Fig. 1b). However, the amplitude of mIPSCs in the Mg^2+^-free group decreased compared with that in the control (*p* < 0.05, Fig. 1b). Reduced mIPSC amplitude is related to reduced number of post-synaptic GABAaRs.

Western blotting demonstrated that the total protein expression of GABRA1 in the Mg^2+^-free group did not change significantly compared with that in the control (*p* > 0.05, Fig. 1c). To investigate the potential role of inhibitory neurotransmitters in this model, GABA vesicular transporter (VGAT), an indicator of GABA release [46], was examined. There were no significant changes in VGAT in the Mg^2+^-free group compared with the control group (*p* > 0.05, Fig. 1c). However, the expression of GABRA1 on the cell surface was significantly decreased in the Mg2 + -free group compared with the control group (*p* < 0.01, Fig. 1d).

To further verify changes in GABRA1 and VGAT in vivo, we constructed a PTZ-induced SE model. In the in vivo model*,* no significant seizures or epileptic discharges were observed on EEG in the control group (Fig. 1e). In contrast, rats in the SE group exhibited significant seizures of stages 4 and above. EEG revealed recurrent interictal epileptic discharges. Moreover, generalized multispike waves and multiple spike and slow-wave complexes were recorded on EEG during the ictal period (Fig. 1e). Western blotting revealed that the total protein expression levels of GABRA1 and VGAT in the SE group did not change significantly (*p* > 0.05, Fig. 1f). In the SE group, the expression of GABRA1 on the cell surface was significantly decreased in comparison to the control group (*p* < 0.05, Fig. 1g).

The data from both the in vitro and in vivo models mentioned above suggest that reduced α1-containing GABAaRs on the neuronal surface may be a key factor in seizures. To further investigate whether decreased surface GABAaR level is associated with receptor internalization, we performed an internalization assay. The intracellular accumulation ratio of GABRA1 increased in the Mg^2+^-free group compared with the control group (intracellular accumulation ratio _Crtl_ = 0.23 ± 0.02, intracellular accumulation ratio_Mg2+-free_ = 0.70 ± 0.04, *p* < 0.01, Fig. 1h). These results suggest that the internalization of α1-containing GABAaRs could be enhanced in seizures, which may be the factor leading to the reduction of α1-containing GABAaRs on the cell surface.

### NPTN plays an antiepileptic role in seizures via increasing the expression of surface α1-containing GABAaRs

**Fig. 2:**
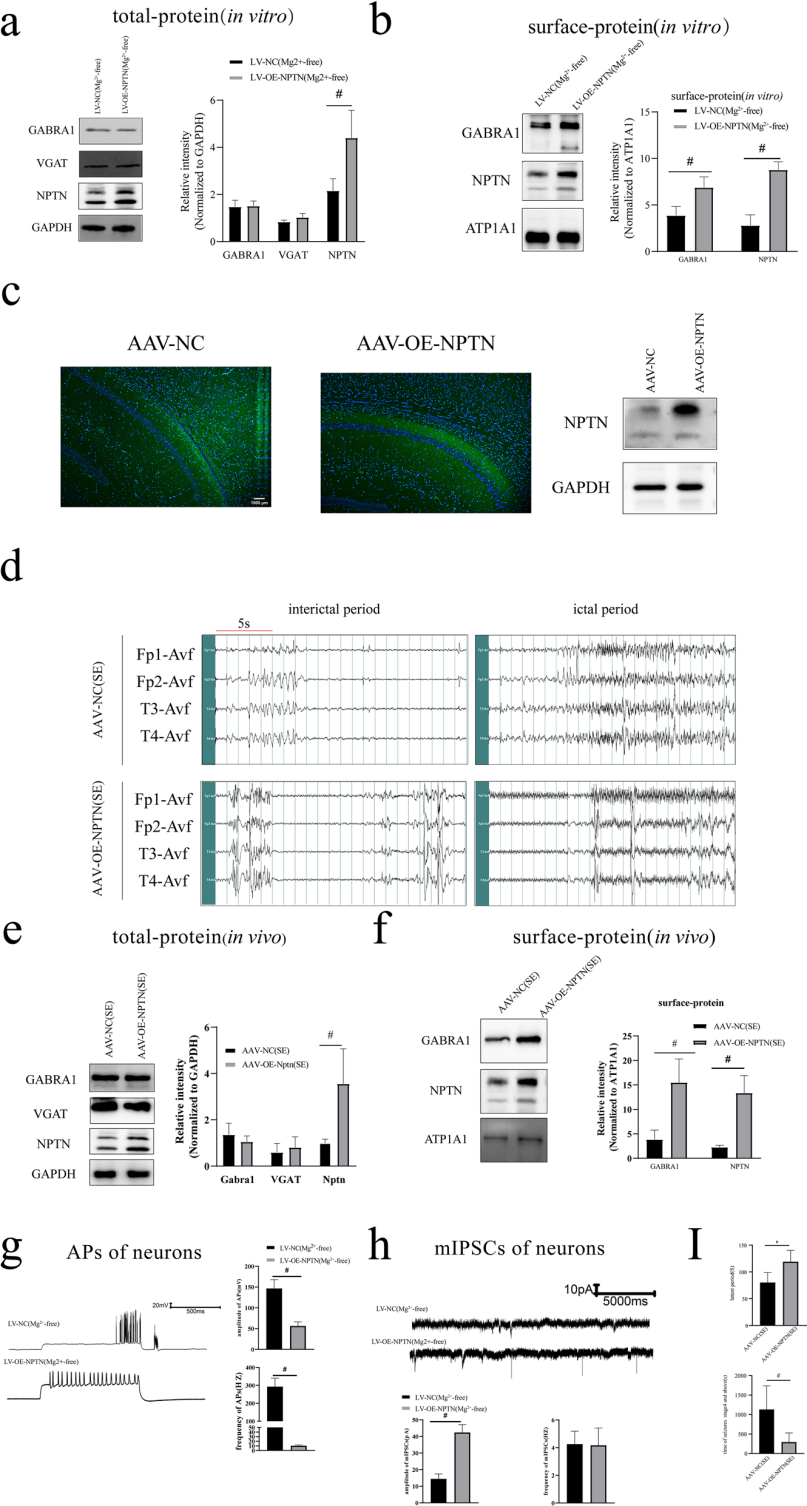
**a** Total protein expression (in vitro, transfected with LV): NPTN expression in the LV-OE-NPTN (Mg2 + -free) group was increased compared with that in the LV-NC (Mg2 + -free) group (*n* = 5 in each group, #*p* < 0.01), while changes in GABRA1 and VGAT protein expression were not observed. **b** Surface protein (in vivo, transfected with LV): Surface GABRA1 protein expression was increased in the LV-OE-NPTN (Mg2 + -free) group compared with the LV-NC (Mg2 + -free) group (*n* = 5 in each group, #*p* < 0.01).** c** Spontaneous green fluorescence in the hippocampus of rats in each group randomly selected for paraffin sectioning after AAV transfection (× 100). Scale bar:1000 μm. The AAV-OE-NPTN group showed an increasing trend of NPTN expression in the hippocampus of AAV-transfected rats. **d** EEG recording showed recurrent epileptic discharges during the interictal period and generalized multi-spike waves and multi-spike waves complexes during the ictal period in both the AAV-NC (SE) and AAV-OE-NPTN (SE) groups. time scale**:** 5 s**. e** Total protein expression (in vivo, transfected with AAV): NPTN protein expression in the AAV-OE-NPTN (SE) group was increased compared with that in the AAV-NC (SE) group (*n* = 5 in each group, #*p* < 0.01), while GABRA1 and VGAT protein expression did not change. **f** Surface protein (in vivo, transfected with AAV): Surface GABRA1 protein expression was increased in the AAV-OE-NPTN(SE) group compared with the AAV-NC (SE) group (*n* = 5 in each group, #*p* < 0.01). **g** APs of neurons transfected with LV: AP frequency was decreased in the LV-OE-NPTN group compared with the LV-NC (Mg2 + -free) group (*n* = 6 in each group, #*p* < 0.01). AP amplitude in the LV-OE-NPTN (Mg2 + -free) group did not change significantly. **h** mIPSCs of neurons transfected with LVs: The frequency of mIPSCs in the LV-OE-NPTN (Mg2 + -free) group did not change significantly, while the amplitude of mIPSCs in the LV-OE-NPTN (Mg2 + -free) group increased compared with that in the LV-NC (Mg2 + -free) group (*n* = 6 in each group, #*p* < 0.01).** i** Latent period and duration of seizures: The latent period from PTZ injection to the first tonic-clonic seizure was 80.20 ± 18.55 s in the AAV-NC (SE) group and 119.00 ± 21.45 s in the AAV-OE-NPTN group (SE) (*n* = 5 in each group, *p < 0.05). Seizure duration (stage 4 and above) was 1128 ± 609.35 s in the AAV-NC (SE) group and 291.80 ± 235.00 s in the AAV-OE-NPTN (SE) group (*n* = 5 in each group, #*p* < 0.01). All experiments were repeated for 3 times. ^△^Independent t-tests were used for the comparison of means.

Western blotting results showed that the total protein expression of NPTN was significantly decreased in both the Mg2^+^-free (*p* < 0.01, Fig. 1c) and SE (*p* < 0.01, Fig. 1f) groups compared with the control group. The surface protein expression of NPTN was significantly decreased in both the Mg2^+^-free (*p* < 0.01, Fig. 1d) and SE (*p* < 0.01, Fig. 1g) groups compared with the control group. Similarly, the expression of GABRA1 on the neuronal surface showed a downward trend in both groups (Fig. 1d and g). To further investigate the correlation between GABRA1 and NPTN, we overexpressed NPTN using the LV vector in neurons the AAV vector in rats. Primary neurons were transfected with LVs, including LV-NC and LV-OE-NPTN. Transfected neurons were exposed to Mg^2+^-free extracellular fluid for 3 h. We found that the total and surface protein expression of NPTN was significantly increased in the LV-OE-NPTN (Mg^2+^-free) group compared with the LV-NC (Mg^2+^-free) group (*p* < 0.01, Fig. 2a;* p* < 0.01, Fig. 2b). Moreover, the expression of GABRA1 on the neuronal surface was significantly increased in the LV-OE-NPTN group compared with the LV-NC (Mg^2+^-free) group (*p* < 0.01, Fig. 2b). However, the total protein expression levels of GABRA1 and VGAT did not change significantly (Fig. 2a). We used an AAV vector to construct NPTN-overexpressing rats. The rats were divided into an AAV-NC group and an AAV-OE-NPTN group. To verify transfection efficiency, two rats from each group were randomly selected after 4 weeks to study spontaneous fluorescence and NPTN expression in the hippocampus. Green fluorescence was noted in the hippocampus 4 weeks after AAV transfection (Fig. 2c), demonstrating successful stereotactic injection of AAV into the hippocampus. The total protein expression of NPTN in the AAV-OE-NPTN group was higher than that in the AAV-NC group, providing evidence of successful NPTN overexpression (Fig. 2c). PTZ-induced SE models were established for both groups. The AAV rats in both groups exhibited recurrent seizures after PTZ administration. All rats were characterized by significant seizures of Stage 4 and above. EEG showed recurrent epileptic discharges during the interictal period, and generalized multi-spike waves and multi-spike-slow wave complexes were recorded during the ictal period in both the AAV-OE-NPTN (SE) and AAV-NC (SE) groups (Fig. 2d). Western blotting results showed that the total and surface protein expression of NPTN was higher in the AAV-OE-NPTN (SE) group than in the AAV-NC (SE) group *(p* < 0.01, Fig. 2e; *p* < 0.01, Fig. 2f). Notably, the expression of GABRA1 on the neuronal surface was significantly increased in the AAV-OE-NPTN (SE) group compared with the AAV-NC (SE) group, (*p* < 0.01, Fig. 2f). However, the total VGAT protein expression did not significantly change in the AAV-OE-NPTN (SE) group compared with the AAV-NC (SE) group (*p* > 0.05, Fig. 2e).

The abovementioned results show a positive correlation between NPTN and surface GABRA1 expression in neurons. Electrophysiological and behavioral recordings were performed to explore the function of NPTN in seizures. The amplitude of APs in the LV-OE-NPTN (Mg^2+^-free) group change significantly compared with that in the LV-NC (Mg^2+^-free) group* (p* < 0.01, Fig. 2 g). Furthermore, the frequency of APs was significantly decreased in the LV-OE-NPTN (Mg^2+^-free) group compared with the LV-NC (Mg^2+^-free) group (*p* < 0.01, Fig. 2 g). The amplitude of mIPSCs in the LV-OE-NPTN (Mg^2+^-free) group was significantly increased compared with that in the LV-NC (Mg^2+^-free) group (*p* < 0.01, Fig. 2 h). However, the frequency of mIPSCs in the LV-OE-NPTN group did not significantly change compared with that in the LV-NC(Mg^2+^-free) group (*p* > 0.05, Fig. 2 h). Moreover, rats in the AAV-OE-NPTN (SE) group had a longer latent period of the first seizure than those in the AAV-NC (SE) group (*p* < 0.05, Fig. 2i), and they also had a shorter duration of seizures than those in the AAV-NC (SE) group, which were all stage 4 and above (*p* < 0.01, Fig. 2i).

These results from both in vitro and in vivo models mentioned above strongly suggest that NPTN inhibits synchronized discharges of neurons and exerts an antagonistic effect in seizures via increasing the expression of α1-containing GABAaRs on the neuronal surface.

### NPTN can inhibit internalization of α1-containing GABAaRs by binding to GABRA1

**Fig. 3:**
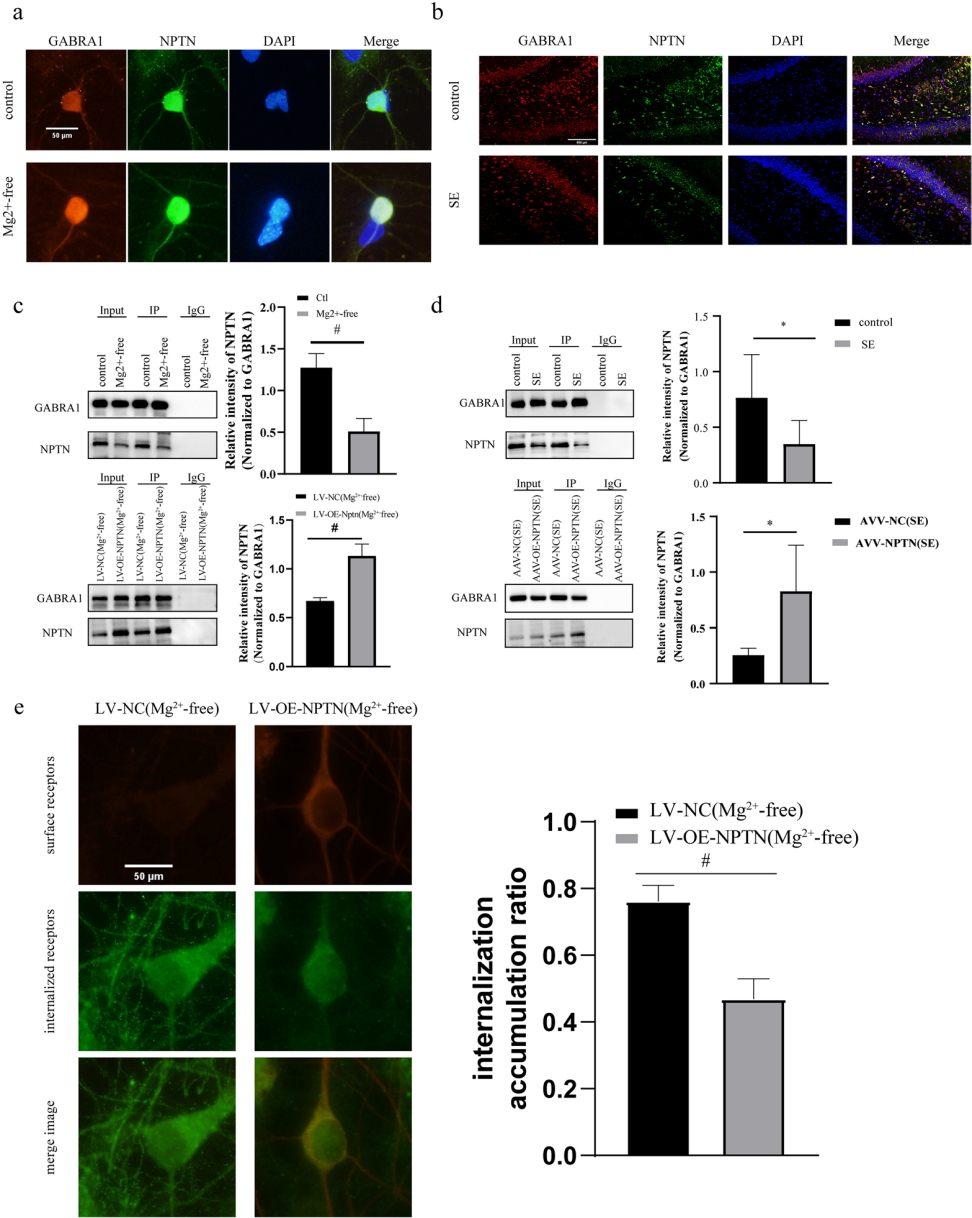
GABRA1 and NPTN interaction in the primary neurons and hippocampus tissues of rats and GABRA1 endocytosis **a** Immunofluorescence co-localization analysis of primary neurons (microscopes, Olympus BX53; objectives, neurons; time of exposure, 50 ms; optic/digital magnification conditions, × 600): GABRA1 co-localized with NPTN in neurons (PCC_Ctrl_ = 0.83, PCC_Mg2+-free_ = 0.91). Scale bar: 50 μm. **b** Immunofluorescence co-localization analysis of hippocampus tissue (microscopes, Olympus BX53; objectives, hippocampal tissues; time of exposure, 250 ms; optic/digital magnification conditions, × 200): GABRA1 co-localized with NPTN in hippocampus tissue (PCC _Ctrl_ = 0.61, PCC_SE_ = 0.73). Scale bar: 500 μm. **c** Co-IP *(*in vitro): Pooled quantification of protein immunoprecipitation shows a significant reduction in the pull-down of NPTN in the Mg2 + -free group compared with the control group (*n* = 6 in each group, #*p* < 0.01) and significant increase in the pull-down of NPTN in the LV-OE-NPTN (Mg2 + -free) group compared with the LV-NC (Mg2 + -free) group (*n* = 5 in each group, #*p* < 0.05). **d** Co-IP (in vivo): Pooled quantification of protein immunoprecipitation shows a significant reduction in the pull-down of the NPTN in the SE group compared with the control group (*n* = 6 in each group, *p < 0.05) and a significant increase in the pull-down of NPTN in the AAV-OE-NPTN (SE) group compared with the AAV-NC (SE) group (*n* = 5 in each group, *p < 0.05). **e** The intracellular accumulation ratio of neurons in the LV-OE-NPTN (Mg2 + -free) group was decreased significantly compared with that of those in the control group (intracellular accumulation ratio _LV-NC (Mg2+-free)_ = 0.76 ± 0.05, intracellular accumulation ratio _LV-NC (Mg2+-free)_ = 0.47 ± 0.06, *n* = 9 in each group, #*p* < 0.01). Scale bar: 50 μm. All experiments were repeated for 3 times. ^△^Independent t-tests were used for the comparison of means

To explore whether there is an interaction between GABRA1 and NPTN, we performed immunofluorescence co-localization analysis. We found that GABRA1 co-localized with NPTN in both primary neurons (PCC_Ctrl_ = 0.83, PCC_Mg2+-free_ = 0.91, Fig. 3a) and hippocampal tissue (PCC _Ctrl_ = 0.61, PCC_SE_ = 0.73, Fig. 3b).

To further demonstrate the binding between GABRA1 and NPTN, natural proteins were extracted from primary neurons and hippocampal tissues using Co-IP. NPTN was expressed as a pull-down protein in both primary neurons (Fig. 3c) and hippocampal tissues (Fig. 3d). Pooled quantification of protein immunoprecipitation shows a significant reduction in the pull-down of NPTN in both the Mg2 + -free (*p* < 0.01, Fig. 3c) and SE groups (*p* < 0.05, Fig. 3d) when compared with the control group. Pooled quantification of protein immunoprecipitation shows a significant increases in the pull-down of NPTN in both the LV-OE-NPTN (Mg2 + -free) group compared with the LV-NC (Mg2 + -free) group (*p* < 0.01, Fig. 3c) and in the AAV-OE-NPTN (SE) group compared with the AAV-NC (SE) group (*p* < 0.05, Fig. 3d).These results demonstrate a reduced number of formed complexes of GABRA1 and NPTN during seizures.

To assess whether the internalization of α1-containing GABAaRs was regulated by NPTN, the internalization assay was performed. The intracellular accumulation ratio of neurons in the LV-OE-NPTN (Mg^2+^-free) group decreased significantly when compared with that of those in the LV-NC (Mg^2+^-free) group (intracellular accumulation ratio _LV-NC (Mg2+-free)_ = 0.76 ± 0.05, intracellular accumulation ratio _LV-NC (Mg2+-free)_ = 0.47 ± 0.06, *p* < 0.01, Fig. 3e). These results suggest that NPTN could inhibit the internalization of α1-containing GABAaRs by binding to GABRA1.

## Discussion

### E/I imbalance is associated with the internalization of α1-containing GABAaRs

The mechanism of seizures is associated with an E/I imbalance, which is attributed to the strengthening of excitatory synapses [6] and/or weakening of inhibitory synapses [8]. The function of the inhibitory synapses is closely related to the number of GABAergic receptors on the neuronal surface. Mounting evidence indicates that a decrease in the number of GABAaRs on the surface of neurons plays a pivotal role in seizure development [47]. Neurons can be induced into synchronized discharge with Mg^2+^-free extracellular solutions [35]. Shorter exposures (< 3 h) to Mg^2+^-free extracellular solution was not consistently shown to produce permanent alterations in neuronal excitability [48]. When the exposure time reached 3 h, we immediately recorded neuronal electrophysiological activity. Our results showed that the amplitude and frequency of APs in the Mg^2+^-free group were significantly increased, which indicated neuronal hyperexcitation. These results were similar to those of Goodkin [35] and Blair [47]. To further investigate the inhibitory function of neurons, we measured their mIPSCs. The frequency of mIPSCs is mainly influenced by GABA release, whereas the amplitude of mIPSCs is influenced by the number of postsynaptic GABAaRs [49]. In our study, we found that the amplitude of mIPSCs was decreased in the Mg2 + -free group, which was related to a reduced number of post-synaptic GABAaRs, whereas the frequency of mIPSCs was not significantly changed. Moreover, the expression of GABRA1 on the neuronal surface decreased in the Mg^2+^-free group, and the change in VGAT expression that reflects neuronal GABA release was not evident, which was consistent with the results of the neuron mIPSC detection. In the PTZ-induced SE model, we also found that GABRA1 expression on the neuronal surface was decreased, and the change in VGAT was not evident. These results indicate that the decrease in α1-containing GABAaRs on the neuronal surface is closely related to acute seizures. Sun et al. suggested that the amplitude of mIPSCs in the hippocampus mainly decreases within 60 min of an acute seizure, and the frequency of mIPSCs gradually decreases after 60 min [50]. The release of GABA neurotransmitters is mainly related to the duration after seizures, which may explain why we did not observe a change in VGAT.

GABAaRs are first assembled in the endoplasmic reticulum and then undergo palmitoylation once transported to the Golgi body, which enhances the hydrophobicity of proteins and promotes the translocation of receptors through the Golgi apparatus to the plasma membrane and synapses [51]. Previous reports have shown that the number and location of GABAaRs on the cell membrane keep changing [51] and can be dynamically regulated through neuronal excitability processes, including movement [52], internalization [35], and recycling [53, 54] of the receptors. These processes are also referred to as GABAaR dynamics. The interactions between the α subunits of GABAaR and postsynaptic scaffold proteins promote GABAaR accumulation on the postsynaptic membrane, forming clusters [55]. The potential lateral spread of postsynaptic GABAaR clusters to the nonsynaptic plasma membrane is related to the phosphorylation of postsynaptic scaffold proteins [56, 57]. The decrease in the interaction between α subunits and postsynaptic scaffold proteins leads to GABAaRs leaving the postsynaptic membrane and triggering the dephosphorylation of β and γ subunits and mechanisms of AP2/clathrin/dynamin, which ultimately leads to the internalization of the GABAaRs [4]. Internalized receptors can be transported to the plasma membrane with the help of kinesins or degraded by lysosomes [36]. To further investigate whether the internalization of GABAaRs in neurons is involved in the pathogenesis of seizures, we performed the internalization assay of α1-containing GABAaRs. The internalization assay suggested that enhanced internalization of α1-containing GABAaRs is associated with excitatory discharge of neurons. Therefore, these data further suggest that enhanced internalization of α1-containing GABAaRs is one of the key factors leading to the decrease of α1-GABAaRs on the cell surface, which is associated with seizures.

### NPTN exerts an antiepileptic effect in seizures by regulating α1-containing GABAaRs on neuron surface

Our results suggest that surface GABRA1 in neurons plays a key role in seizures. The interaction between NPTN and GABRA1 demonstrated the highest PPI prediction score. NPTN is mostly clustered at synapses in the central nervous system and belong to the CAM family [58]. NPTN plays an important role in the E/I balance by regulating neurotransmitter receptors, such as GABAaRs, NMDARs, and AMPARs [23, 33, 34, 59, 60]. Our results showed that the expression of NPTN decreased in both the Mg^2+^-free and SE groups*.* Similarly, the surface GABRA1 of the neurons showed a downward trend in both groups. Next, we constructed in vitro and in vivo models of NPTN overexpression via transfection of primary neurons with LVs and hippocampal injection of AAVs. The transfected neurons were exposed to a Mg^2+^-free solution for 3 h, and the transfected rats were exposed to PTZ to induce SE. Western blotting showed that GABRA1 on the neuronal surface increased in the LV-OE-NPTN (Mg2 + -free) and AAV-OE-NPTN (SE) groups. These results suggest that NPTN expression is positively correlated with surface GABA1 expression in neurons.

To further investigate the function of NPTN in seizures, we evaluated the electrophysiology of the transfected neurons exposed to Mg^2+^-free extracellular fluid. Neurons in the LV-OE-NPTN (Mg^2+^-free) group showed a lower frequency of APs than those in the LV-NC group (Mg^2+^-free). In addition, the mIPSC results showed that the amplitude in the LV-OE-NPTN (Mg^2+^-free) group increased, suggesting that NPTN could promote the number of GABAaRs in the postsynaptic membrane, which could inhibit synchronized discharges of neurons*.* In an in vivo model, we found that the latent period was longer and the duration of stage 4 and above seizures was shorter in the AAV-OE-NPTN (SE) group. These results confirmed the antiepileptic function of NPTN. However, the upregulation of NPTN did not cause any significant change in VGAT expression or in the frequency of mIPSCs in the LV-OE-NPTN (Mg2 + -free) group, suggesting that NPTN mainly regulates α1-containing GABAaRs on the surface of neurons. Therefore, our results suggest that NPTN exerts an antiepileptic effect in seizures by regulating α1-containing GABAaRs on neuron surface. In our study, we have also constructed NPTN-deficient rats and no spontaneous seizures were observed. However, the NPTN-deficient rats could not complete the following experiments due to insufficient survival time after injecting PTZ. Previous studies also showed that the mice which was lack of Np65 (an isoform of NPTN) did not exhibit spontaneous epilepsy [61, 62], but exhibited significant neurotoxicity in stroke models, which indicating that NPTN-deficient mice can exhibit significant E-I imbalance by receiving proper exogenous stimulation [61]. Since NPTN is not the only anchor protein of GABAaRs [22], simple deficiency of NPTN will not cause spontaneous seizures. Therefore, we infer that NPTN deficiency is more likely to increase the susceptibility to seizure.

In addition to increasing the amount of a1-GABAaRs on the surface of neurons, NPTN may also increase α2-containing GABAaRs on the surface of neurons [34], which may also play an antiepileptic role. However, the regulation mechanism of NPTN on a2-GABAaRs still needs to be further studied. NPTN may also play an antiepileptic role by inhibiting neuronal overexcitation. Ruth M. Empson et al. found that NPTN reduced surface expression of AMPARs [23]. Yuhui Hu et al. suggested that the vesicular glutamate transporter-1(VGluT1), and NMDARs were significantly increased in NPTN-KO mice [61]. Excessive accumulation of calcium ions in neurons is associated with seizures [63]. NPTN is the obligatory auxiliary subunits of native PMCAs and key regulator of intracellular Ca2 + clearance [64, 65].

### NPTN inhibits GABAaR internalization via binding to GABRA1

Our results suggested that there is a positive correlation between NPTN and surface GABRA1 in neurons and that the antiepileptic effects of NPTN may be related to the promotion of GABRA1 expression on the cell surface. NPTN belongs to the CAM family of proteins [23], which has some members that are located on postsynaptic membranes of neurons, including cadherins, neuroligins, and neurexins [66–68]. Previous studies have shown that these proteins stabilize GABAaRs on the neuronal surface, primarily by binding to receptor subunits [66–68]. These proteins may also contribute to the pathogenesis of epilepsy by influencing the E/I balance [69, 70]. Therefore, we proposed a hypothesis that NPTN may have a function similar to CAM, and could thus stabilize the GABAaRs on the neuronal surface. To further demonstrate the relationship between GABRA1 and NPTN, immunofluorescence co-localization analysis was performed. We found that GABRA1 co-localized with NPTN both in the primary neurons and hippocampus tissue, which is consistent with the experimental results of Herrera-Molina et al. [33]. To further investigate the regulatory mechanism of NPTN on α1-containing GABAaRs, we used Co-IP assay to show that NPTN binds to GABRA1 to form protein complexes. The interaction between NPTN and GABRA is associated with abnormal neuronal excitation. In the Mg^2+^-free and PTZ-induced SE models, the expression of NPTN decreased; correspondingly, the interaction between NPTN and GABRA1 was weakened. In contrast, the number of complexes formed of NPTN and GABRA1 was increased after overexpression of NPTN. To investigate the potential mechanism by which NPTN promotes the increase in GABRA1 on the cell surface, we performed an internalization assay in primary neurons transfected with LV. Finally, we confirmed that NPTN effectively inhibited the internalization of GABRA1.

These observations collectively suggest that NPTN exerts antiepileptic effects through binding to the α1 subunit of GABA type A receptor to inhibit the internalization of these receptors. Most seizures are time-limited events that do not require emergency intervention to terminate [71]. The exceptions are prolonged seizures, which can have serious consequences [2, 3]. Previous clinical trials and guidelines suggest prolonged seizures should be terminated within 5 min [72, 73]. Currently, the main way to terminate a seizure is to activate GABAaRs on the neuronal surface through benzodiazepines [18]. Nevertheless, benzodiazepines have been shown to be able to terminate seizures in only 43–89% of patients [74]. Patients who are insensitive to benzodiazepines are more likely to develop refractory SE, which is closely related to GABAaR internalization [75]. In addition to the high risk of mortality, refractory SE can have long-term consequences, including neuronal death, neuronal injury, and alteration of neuronal networks [3]. The enhanced internalization of GABAaRs leads to a reduction in the action sites of benzodiazepines on the neuron membrane, resulting in synchronized discharges of neurons and difficult termination of the discharges [76]. Meanwhile, the synchronized discharge of neurons increases the internalization of GABAaRs [35]. In the face of these challenges, our findings provide evidence that NPTN could be a new target for seizure termination. Furthermore, NPTN can be synergistic with benzodiazepines in the treatment of refractory SE by promoting the sensitivity of benzodiazepines through increasing the amount of GABAaR on the neuronal surface, thereby preventing the occurrence of refractory SE.

Nevertheless, our study has some limitations. The binding sites of NPTN and GABRA1 and protein modification mechanism of NPTN have not yet been elucidated. Future studies are warranted to unravel these protein bindings and modifications that govern the NPTN-GABRA1 interactions and their antiepileptic effects.

## Conclusion

This is the first study reporting the mechanisms of NPTN in seizures. NPTN expression was found to be positively correlated with GABRA1 expression of the neuronal surface. The expression of GABRA1 was found to be decreased on the neuronal surface which was associated with increased internalization of α1-containing GABAaRs in seizure models. Our findings provide evidence that NPTN may exert antiepileptic effects by binding to GABRA1 to inhibit the internalization of α1-containing GABAaRs.

### Supplementary Information

**Additional file: 1**. Specificity of anti-neuroplastin antibody.

## Data Availability

The data supporting the findings of this study are available from the corresponding author upon request.

## Abbreviations

AAV: Adeno-associated virus
AP: Action potential
ARHGEF9: Rho guanine nucleotide exchange factor 9
BSA: Bovine serum albumin
CAM: Cell adhesion molecule
Co-IP: Co-immunoprecipitation
EEG: Electroencephalography
GABAaRs: γ-Aminobutyric type A acid receptors
GABRA1: GABAaR α1 subunit
GPN: Gephyrin
LV: Lentivirus
mIPSC: Miniature inhibitory postsynaptic currents
NC: Nitrocellulose
NPTN: Neuroplastin
PBS: Phosphate-buffered saline
PTZ: Pentylenetetrazol
SD: Sprague–Dawley
SE: Status epilepticus
SDS-PAGE: Sodium dodecyl-sulfate polyacrylamide gel electrophoresis
VGAT: GABA vesicular transporter
