# Supplementary figures and images for: Neuroplastin exerts antiepileptic effects through binding to the α1 subunit of GABA type A receptors to inhibit the internalization of the receptors
Source: J Transl Med. 2023 Oct 9;21:707. doi: 10.1186/s12967-023-04596-4 (PMC10563248; doi:10.1186/s12967-023-04596-4)

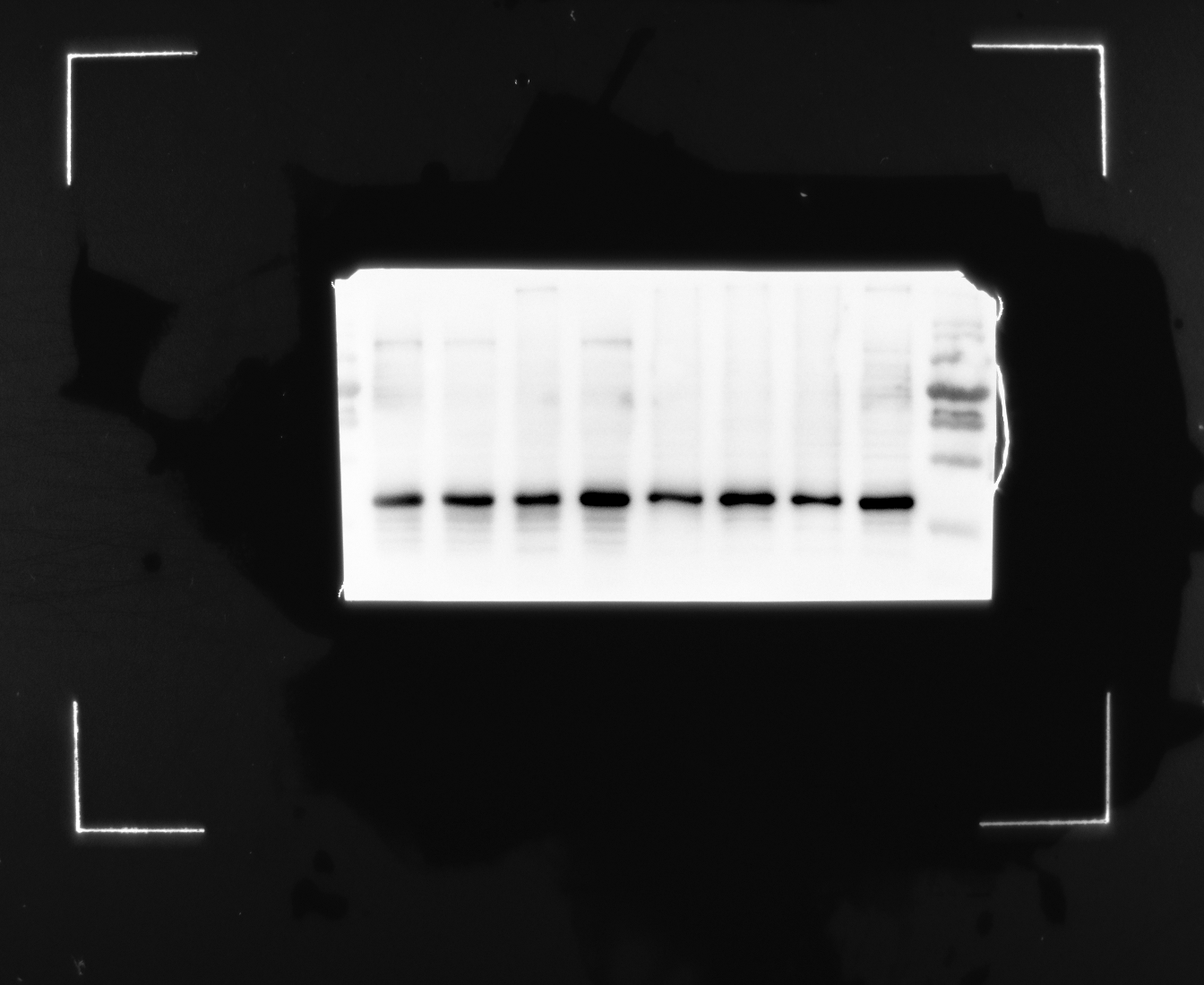

Supplement: Supplementary file 1 — Additional file: 1. Specificity of anti-neuroplastin antibody. [file 12967_2023_4596_MOESM1_ESM.zip › ESM/GAPDH for NPTN-RNAi-neuron .tif]

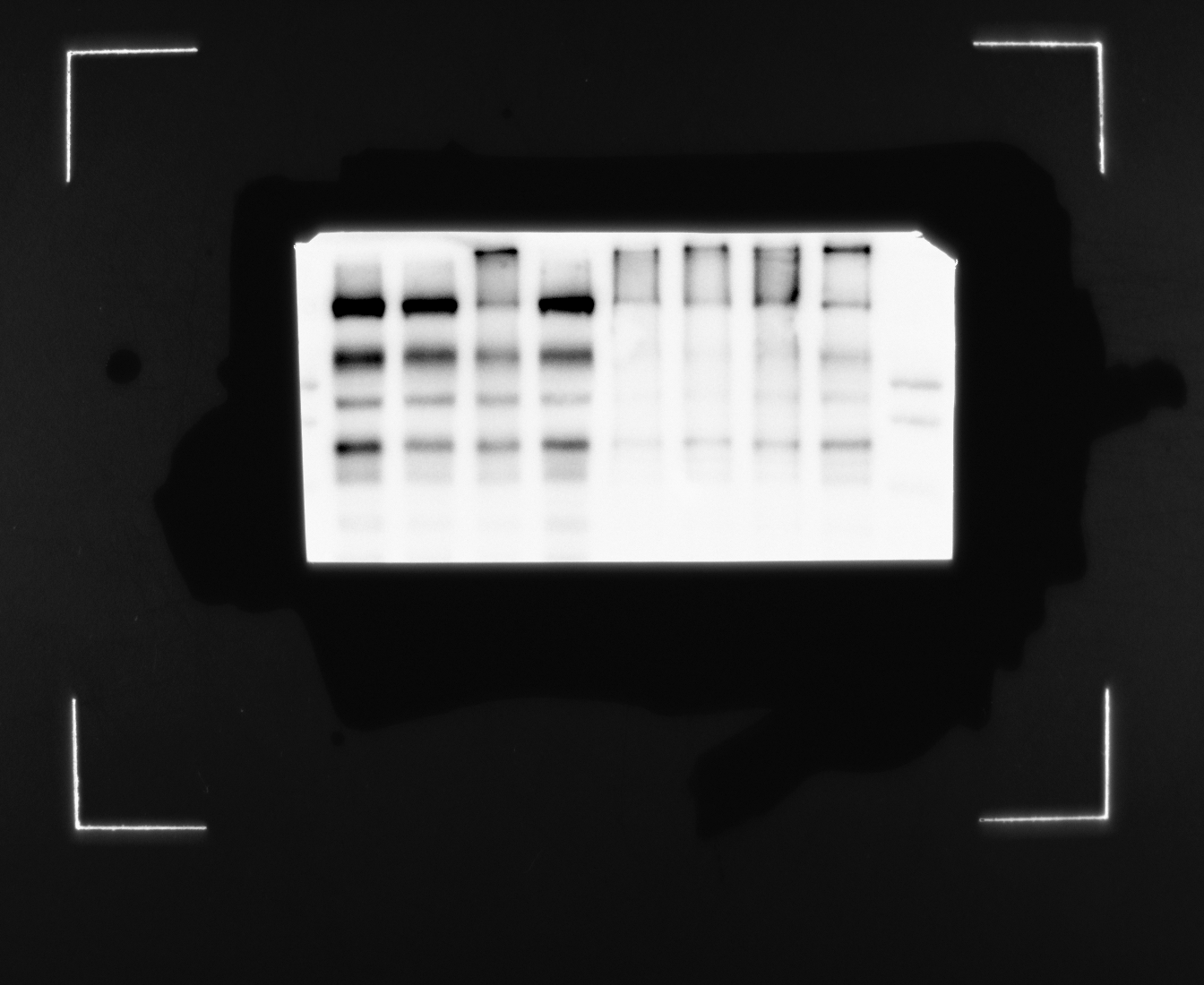

Supplement: Supplementary file 1 — Additional file: 1. Specificity of anti-neuroplastin antibody. [file 12967_2023_4596_MOESM1_ESM.zip › ESM/NPTN-RNAi-neuron.tif]
